# Air pollution and survival in patients with malignant mesothelioma and asbestos-related lung cancer: a follow-up study of 1591 patients in South Korea

**DOI:** 10.1186/s12940-024-01094-y

**Published:** 2024-06-10

**Authors:** Da-An Huh, Yun-Hee Choi, Lita Kim, Kangyeon Park, Jiyoun Lee, Se Hyun Hwang, Kyong Whan Moon, Min-Sung Kang, Yong-Jin Lee

**Affiliations:** 1 Institute of Health Sciences, Korea University, Anam-ro 145, Seongbuk-gu, Seoul, 02841 South Korea; 2 Department of Ophthalmology, Korea University College of Medicine, Anam-ro 145, Seongbuk- gu, Seoul, 02841 South Korea; 3 Department of Health and Safety Convergence Science, Korea University, Anam-ro 145, Seongbuk-gu, Seoul, 02841 South Korea; 4 L-HOPE Program for Community-Based Total Learning Health Systems, Korea University, Anam-ro 145, Seongbuk-gu, Seoul, 02841 South Korea; 5 School of Health and Environmental Science, Korea University, Anam-ro 145, Seongbuk-gu, Seoul, 02841 South Korea; 6 Environmental Health Center for Asbestos, Soonchunhyang University Cheonan Hospital, 31 Suncheonhyang 6-gil, Dongnam-gu, Cheonan-si, 31151 South Korea; 7 Department of Occupational & Environmental Medicine, Soonchunhyang University, 31 Suncheonhyang 6-gil, Dongnam-gu, Cheonan-si, 31151 South Korea

**Keywords:** Air pollution, Exposure mixtures, Lung cancer, Malignant mesothelioma, Survival analysis

## Abstract

**Background:**

Despite significant advancements in treatments such as surgery, radiotherapy, and chemotherapy, the survival rate for patients with asbestos-related cancers remains low. Numerous studies have provided evidence suggesting that air pollution induces oxidative stress and inflammation, affecting acute respiratory diseases, lung cancer, and overall mortality. However, because of the high case fatality rate, there is limited knowledge regarding the effects of air pollution exposures on survival following a diagnosis of asbestos-related cancers. This study aimed to determine the effect of air pollution on the survival of patients with malignant mesothelioma and asbestos-related lung cancer.

**Methods:**

We followed up with 593 patients with malignant mesothelioma and 998 patients with lung cancer identified as asbestos victims between 2009 and 2022. Data on five air pollutants—sulfur dioxide, carbon monoxide, nitrogen dioxide, fine particulate matter with a diameter < 10 μm, and fine particulate matter with a diameter < 2.5 μm—were obtained from nationwide atmospheric monitoring stations. Cox proportional hazard models were used to estimate the association of cumulative air pollutant exposure with patient mortality, while adjusting for potential confounders. Quantile-based g-computation was used to assess the combined effect of the air pollutant mixture on mortality.

**Results:**

The 1-, 3-, and 5-year survival rates for both cancer types decreased with increasing exposure to all air pollutants. The estimated hazard ratios rose significantly with a 1-standard deviation increase in each pollutant exposure level. A quartile increase in the pollutant mixture was associated with a 1.99-fold increase in the risk of malignant mesothelioma-related mortality (95% confidence interval: 1.62, 2.44). For lung cancer, a quartile increase in the pollutant mixture triggered a 1.87-fold increase in the mortality risk (95% confidence interval: 1.53, 2.30).

**Conclusion:**

These findings support the hypothesis that air pollution exposure after an asbestos-related cancer diagnosis can negatively affect patient survival.

**Supplementary Information:**

The online version contains supplementary material available at 10.1186/s12940-024-01094-y.

## Introduction

Malignant mesothelioma and lung cancer are representative diseases caused by asbestos exposure [1]. Although the harmful effects of asbestos have become widely known, leading to its ban in many countries, cases of asbestos-related diseases continue to be reported worldwide owing to its long latency period. In 2019, approximately 35,000 malignant mesothelioma-related deaths occurred globally, which is double the number from 1990 [2]. Additionally, approximately 180,000 deaths from lung cancer worldwide each year are estimated to be caused by asbestos exposure [3].

South Korea was a major consumer nation of asbestos during the 1990s, producing or importing approximately 2–2.4 million tons of asbestos until its use was banned in 2009 [4]. Consequently, cases of malignant mesothelioma and lung cancer owing to occupational and environmental asbestos exposure continue to emerge annually. The Ministry of Environment of South Korea enacted the Asbestos Injury Relief Act in 2011 and established two Environmental Health Centers for Asbestos to operate a health surveillance system [4]. As of 2022, 694 cases of malignant mesothelioma and 1,080 cases of asbestos-related lung cancer have been reported in South Korea, according to data released by the Ministry of Environment.

Asbestos-related cancers generally have a poor prognosis, with low survival rates. In particular, the 5-year survival of patients with malignant mesothelioma is less than 5%, and the median survival duration for patients is reported to be 15 months [5, 6]. Despite significant advancements in treatments such as surgery, radiotherapy, and chemotherapy [7], the survival rate for patients with asbestos-related cancer remains low, highlighting the need for new approaches. One potential strategy is to identify and manage factors influencing survival. Although smoking cessation programs and early patient detection through surveillance systems have garnered attention [8, 9], there has been limited research on other determining factors.

One potential factor that could influence the survival duration of patients with asbestos-related cancer is air pollution, which has been designated as a carcinogen by the International Agency for Research on Cancer [10]. Numerous studies have provided evidence suggesting that air pollution induces oxidative stress and inflammation [11], affecting acute respiratory diseases, lung cancer, and overall mortality [12–17]. Considering that inhaled pollutants can potentially promote tumor progression [18,19], air pollution exposure may shorten the survival duration of patients with asbestos-related cancers after diagnosis. There is limited knowledge regarding the effects of air pollution exposures on survival following a diagnosis of asbestos-related cancers. Given that 91% of the world’s population resides in places where air pollution levels exceed the World Health Organization air quality guidelines [20], it is crucial to investigate the effect of air pollution exposure on the exacerbation of symptoms and the survival duration of patients with malignant mesothelioma and asbestos-related lung cancer.

Thus, the aim of this study was to ascertain the contribution of air pollution to the survival of patients with asbestos-related cancers. We conducted a follow-up study on patients with malignant mesothelioma and asbestos-related lung cancer and utilized the nationwide atmospheric monitoring data of South Korea to estimate the patients’ cumulative exposure to air pollution.

## Materials and methods

### Data source and study population

We used information from patients with malignant mesothelioma and asbestos-related lung cancer collected by the South Korea Ministry of Environment and the Soonchunhyang University Cheonan Hospital, an Environmental Health Center for Asbestos in South Korea. The abovementioned institutions collected information on asbestos-exposed victims through three processes. First, since its establishment in 2009, the Environmental Health Center for Asbestos has been conducting health investigations among residents of areas suspected of asbestos exposure. Areas within a 2-km radius of past asbestos exposure sources, such as asbestos mines and asbestos factories, were classified as presumptive exposure areas. People who had lived in these areas for more than 10 years were investigated [4]. The primary investigation, which was a screening test, included a physical examination by physicians, chest radiography, and a survey on the history of asbestos exposure using a structured questionnaire. Participants with abnormal findings in the primary investigation underwent a detailed examination, which included computed tomography and pulmonary function tests. After the investigation, the Center reported the results for patients with suspected asbestos-related diseases to the Ministry of Environment. Second, individuals seeking compensation for diseases caused by asbestos exposure under the Asbestos Damage Relief Act were required to submit information such as asbestos exposure history and medical records to the local government. This information was transmitted to the Ministry of Environment. Third, adults in South Korea were mandated to undergo regular medical examinations under the National Health Insurance Act. Furthermore, medical institutions were required to report suspected cases of asbestos exposure-related damage to the Ministry of Environment.

The South Korea Environmental Industry and Technology Institute, affiliated with the Ministry of Environment, analyzed the collected information and medical findings and classified patients as asbestos victims if a causal relationship between asbestos exposure and the development of the disease was established. We accessed information on asbestos victims identified through these processes and obtained data on 593 patients with malignant mesothelioma and 998 patients with lung cancer recognized as asbestos victims between 2009 and 2022.

### Air pollution assessment

Data on five air pollutants—sulfur dioxide (SO_2_, parts per billion [ppb]), carbon monoxide (CO, ppb), nitrogen dioxide (NO_2_, ppb), fine particulate matter with a diameter < 10 μm (PM_10_, µg/m^3^), and fine particulate matter with a diameter < 2.5 μm (PM_2.5_, µg/m^3^)—were obtained from the 525 nationwide atmospheric monitoring stations operated by the South Korea Ministry of Environment. We calculated 24-h averages for SO_2_, CO, NO_2_, PM_10_, and PM_2.5_ using hourly measurements from these monitoring stations.

For each patient, we assigned the daily means of ambient air pollution exposure from the date of diagnosis to the date of the last follow-up or death using data from a monitoring station in the patient’s residential area. If no monitoring station existed in the patient’s town or if the available data covered less than 75% of the follow-up period, the average was determined using values from stations within the broader administrative region encompassing that town. The distribution of patients with malignant mesothelioma and asbestos-related lung cancer by administrative region is shown in Figure S1.

### Survival outcome

Survival time was determined as the period between the date of cancer diagnosis to the date of death from any cause. As a sensitivity analysis, cancer-specific survival time was calculated as the period between the date of cancer diagnosis to the date of death due to malignant mesothelioma (Korean standard classification of diseases [KCD]-8 code C45) or lung cancer (KCD-8 code C34). If the patient did not die, the survival time was defined as the time from cancer diagnosis to December 31, 2022, the last follow-up date.

### Covariates

In our statistical models that examine the associations between air pollution exposure and the survival time of patients with asbestos-related cancer, we considered potential confounding factors. These included age at diagnosis (continuous), sex (male and female), smoking status (never-smoker, past smoker, current smoker, and unknown), cancer cell type for malignant mesothelioma (epithelioid, sarcomatoid, and biphasic) and lung cancer (adenocarcinoma, squamous cell, small cell, large cell, and others), type of treatment (surgery, radiotherapy, and chemotherapy), and the month of diagnosis (continuous) to control for seasonal variation effects. Additionally, based on recent preliminary research showing variations in patient survival depending on the type of asbestos exposure [21, 22], we adjusted for asbestos exposure modalities (environmental exposure, occupational exposure, and co-exposure).

For the asbestos exposure modalities, well-trained researchers from the Environmental Health Center used structured questionnaires designed by the Ministry of Environment to gather detailed information on lifetime asbestos exposure. Occupational exposure to asbestos was identified when an individual had worked with asbestos fibers for at least a year. The data collection included details such as the name of the workplace, the type of job, duration of employment, and age at first exposure. To reduce information bias in the survey process, responses from participants were cross-referenced with historical records of the workplace’s location and operational period. Environmental exposure to asbestos was characterized by non-occupational contact with airborne asbestos fibers from sources such as mines, industrial sites, and loading areas. The center also gathered data on participants’ living regions, types of exposure sources, proximity to these sources, duration of residence, age at first exposure, and experience with soil cultivation. To ensure the reliability of the provided exposure data, survey answers were checked against historical records of asbestos exposure in Korea maintained by the Ministry of Environment and residential registration documents of the participants. Those with both occupational and environmental asbestos exposures were categorized into the co-exposure group.

### Statistical analysis

We calculated descriptive statistics for patient characteristics, air pollution exposure, and covariates. Median survival time as well as 1-, 3-, and 5-year survival rates were calculated for patients with malignant mesothelioma and lung cancer according to air pollution exposure levels categorized into quartiles.

Cox proportional hazard models were used to estimate the association of cumulative air pollutant exposure with all-cause and cancer-specific mortalities of patients while adjusting for potential confounders. Results were presented as hazard ratios (HRs) and 95% confidence intervals (CIs) for mortality by a 1-standard deviation (SD) increase in SO_2_, CO, NO_2_, PM_10_, and PM_2.5_ components. The assessment of the proportional hazards assumption was conducted by calculating the Pearson correlation coefficient between Schoenfeld residuals and the time of follow-up. Additionally, we evaluated the nonlinear relationships between air pollutants and HRs of mortality by applying restricted cubic splines with three knots (5th, 50th, and 95th percentiles of each pollutant) after adjusting for all covariates.

To investigate potential interactions between air pollutants, we applied both two- and multi-pollutant models. In the two-pollutant models, we assessed the association between each pollutant and the HR of mortality while adjusting for the other pollutants one by one. For the multi-pollutant models, quantile-based g-computation was used to assess the joint effect of the air pollutant mixture on mortality. Quantile-based g-computation is an approach for assessing the joint effect of air pollutant mixtures when the exposure levels of all pollutants simultaneously increase by one quantile. This methodology has the advantage of not requiring the assumption of linearity of exposure effects [23]. A total of 1000 bootstrap replicates were performed to estimate 95% CIs.

All statistical analyses were performed using R version 4.3.0, and quantile-based g-computation was performed using the ‘qgcomp’ package in R. A statistical significance level was set as a two-sided *p*-value < 0.05.

## Results

Descriptive statistics and characteristics of the study population are presented in Table 1. The mean ages at diagnosis for patients with malignant mesothelioma and asbestos-related lung cancer were 62.9 and 65.6 years, respectively, and more than half of the patients were men. Patients with malignant mesothelioma predominantly underwent chemotherapy (52.4%), whereas patients with lung cancer predominantly underwent surgery (48.0%). The median survival times were 1.58 and 2.58 years for patients with malignant mesothelioma and lung cancer, respectively.

**Table 1:** Descriptive statistics and characteristics of patients with malignant mesothelioma and asbestos-related lung cancer

Variables	Malignant mesothelioma (*n* = 593)	Lung cancer (*n* = 998)
Age at diagnosis, mean ± SD (years)	62.9 ± 12.9	65.6 ± 9.4
Male, n (%)	361 (60.9)	632 (63.3)
Year of diagnosis, n (%)		
2009–2012	140 (23.6)	65 (6.5)
2013–2015	135 (22.8)	147 (14.7)
2016–2018	138 (23.3)	288 (28.9)
2019–2022	180 (30.3)	498 (49.9)
Smoking status, n (%)		
Never-smoker	293 (49.4)	442 (44.3)
Past smoker	255 (43.0)	482 (48.3)
Current smoker	7 (1.2)	10 (1.0)
Unknown	38 (6.4)	64 (6.4)
Asbestos exposure modalities, n (%)		
Environmental	304 (51.3)	423 (42.4)
Occupational	214 (36.1)	150 (15.0)
Co-exposure	75 (12.6)	425 (42.6)
Treatment types (multiple choice), n (%)		
Surgery	171 (28.8)	479 (48.0)
Radiotherapy	10 (1.7)	137 (13.7)
Chemotherapy	311 (52.4)	402 (40.3)
Unknown	163 (27.5)	199 (19.9)
Median survival time (years)	1.58	2.58
Air pollution exposures, mean ± SD		
SO_2_ (ppb)	4.04 ± 1.51	3.38 ± 1.01
CO (ppb)	478.14 ± 102.20	412.77 ± 79.51
NO_2_ (ppb)	22.05 ± 7.33	16.55 ± 5.86
PM_10_ (µg/m^3^)	41.21 ± 9.24	35.74 ± 7.43
PM_2.5_ (µg/m^3^)	21.27 ± 4.76	19.56 ± 4.38

SD, standard deviation; ppb, parts per billion

The correlation coefficients for air pollutant exposures ranged from 0.33 to 0.88 for malignant mesothelioma and 0.18 to 0.87 for lung cancer (Figure S2).

Table 2 shows the median survival time as well as the 1-, 3-, and 5-year survival rates for each quartile of air pollution exposure. The association between survival time and levels of air pollution exposure was inconsistent across the two cancer types. For malignant mesothelioma, patients exposed to the highest levels of SO_2_, CO, NO_2_, and PM_10_ had the shortest survival times. This trend was not observed in patients with lung cancer. Nonetheless, the 1-, 3-, and 5-year survival rates for both cancer types decreased with increasing exposure to all air pollutants. Table S1 presents the median survival times and survival rates based on other covariates, excluding air pollution.

**Table 2:** Median survival time as well as 1-, 3-, and 5-year survival rates based on levels of air pollution exposure

Categorized air pollution exposure	Malignant mesothelioma (*n* = 593)				Lung cancer (*n* = 998)			
	Median survival (years)	Survival rate (%)			Median survival (years)	Survival rate (%)		
		1-year	3-year	5-year		1-year	3-year	5-year
SO_2_ (ppb)								
Q1 (1.26–2.77)	1.67	77.9	59.1	53.7	1.79	94.8	86.0	82.4
Q2 (2.77–3.34)	2.08	78.4	57.4	45.3	2.33	93.6	83.1	81.1
Q3 (3.34–4.12)	1.58	65.5	29.1	18.9	4.00	90.8	75.6	70.8
Q4 (4.12–11.89)	1.25	58.8	17.6	9.5	3.58	83.1	60.2	46.6
CO (ppb)								
Q1 (225.71–400.28)	1.33	70.5	47.0	43.0	1.92	93.6	79.6	76.0
Q2 (400.28–463.16)	1.67	73.6	48.6	37.2	3.42	92.0	79.9	74.7
Q3 (463.16–514.48)	2.08	78.4	43.9	32.4	2.38	87.2	74.8	69.2
Q4 (514.48–1041.54)	1.25	58.1	23.6	14.9	3.33	89.6	70.7	61.0
NO_2_ (ppb)								
Q1 (5.12–15.89)	1.58	72.5	47.0	40.9	2.33	92.8	78.4	74.8
Q2 (15.89–20.25)	1.75	72.3	44.6	35.8	2.58	91.6	81.5	74.3
Q3 (20.25–25.59)	1.71	73.0	45.9	35.1	2.83	92.0	76.0	71.2
Q4 (25.59–50.22)	1.33	62.8	25.7	15.5	3.17	85.9	69.1	60.6
PM_10_ (µg/m^3^)								
Q1 (21.33–33.15)	1.25	74.3	58.1	55.4	1.58	94.4	87.2	85.6
Q2 (33.15–38.48)	2.00	73.0	52.7	41.9	2.75	97.2	84.7	80.3
Q3 (38.48–43.56)	1.79	72.3	36.5	22.3	4.17	93.2	79.2	72.8
Q4 (43.56–68.30)	1.25	61.5	16.2	8.1	2.83	77.5	53.8	42.2
PM_2.5_ (µg/m^3^)								
Q1 (10.69–17.97)	1.17	70.9	52.7	49.1	1.67	94.2	86.7	84.2
Q2 (17.97–21.07)	2.42	89.9	69.7	62.4	2.71	97.9	87.5	82.9
Q3 (21.07–24.37)	3.25	77.1	58.7	43.1	3.88	92.5	80.0	75.4
Q4 (24.37–37.01)	1.58	71.6	29.4	16.5	3.58	83.3	61.5	49.8

ppb, parts per billion

After adjusting for covariates, an increase in exposure to all air pollutants was associated with a heightened mortality risk (Table 3). The estimated HRs from the Cox proportional hazards models for both all-cause and cancer-specific mortalities rose significantly with a 1-SD increase in the exposure level of each pollutant. Among patients with malignant mesothelioma, the largest HRs were estimated for SO_2_ (HR = 1.45 [95% CI: 1.32, 1.60]), PM_10_ (HR = 1.47 [95% CI: 1.28, 1.68]), and PM_2.5_ (HR = 1.65 [95% CI: 1.40, 1.95]). For patients with lung cancer, the largest HRs were estimated for PM_10_ (HR = 2.13 [95% CI: 1.86, 2.44]), PM_2.5_ (HR = 1.99 [95% CI: 1.74, 2.28]), and SO_2_ (HR = 1.48 [95% CI: 1.33, 1.66]).

**Table 3:** Adjusted^a^ hazard ratios (HRs) and 95% confidence intervals (CIs) for all-cause and cancer-specific mortalities associated with a 1-standard deviation (SD) increase in air pollutant exposure^b^

Air pollutant	Malignant mesothelioma (*n* = 593)		Lung cancer (*n* = 998)	
	All-cause mortality HR (95% CI)	Cancer-specific mortality HR (95% CI)	All-cause mortality HR (95% CI)	Cancer-specific mortality HR (95% CI)
SO_2_	1.45 (1.32, 1.60)	1.45 (1.32, 1.60)	1.47 (1.31, 1.64)	1.48 (1.33, 1.66)
CO	1.30 (1.15, 1.47)	1.28 (1.13, 1.45)	1.24 (1.11, 1.39)	1.26 (1.13, 1.41)
NO_2_	1.19 (1.07, 1.33)	1.17 (1.05, 1.31)	1.28 (1.15, 1.43)	1.29 (1.16, 1.44)
PM_10_	1.52 (1.33, 1.74)	1.47 (1.28, 1.68)	2.11 (1.85, 2.42)	2.13 (1.86, 2.44)
PM_2.5_	1.70 (1.44, 2.00)	1.65 (1.40, 1.95)	1.96 (1.71, 2.25)	1.99 (1.74, 2.28)

^a^Models were adjusted for age at diagnosis, sex, smoking status, cancer cell type, type of treatment, asbestos exposure modalities, and month of diagnosis.

^b^SD values: For malignant mesothelioma, 1.51 ppb (SO_2_), 102.20 ppb (CO), 7.33 ppb (NO_2_), 9.24 µg/m^3^ (PM_10_), and 4.76 µg/m^3^ (PM_2.5_). For lung cancer, 1.01 ppb (SO_2_), 79.51 ppb (CO), 5.86 ppb (NO_2_), 7.43 µg/m^3^ (PM_10_), and 4.38 µg/m^3^ (PM_2.5_).

**Fig. 1:**
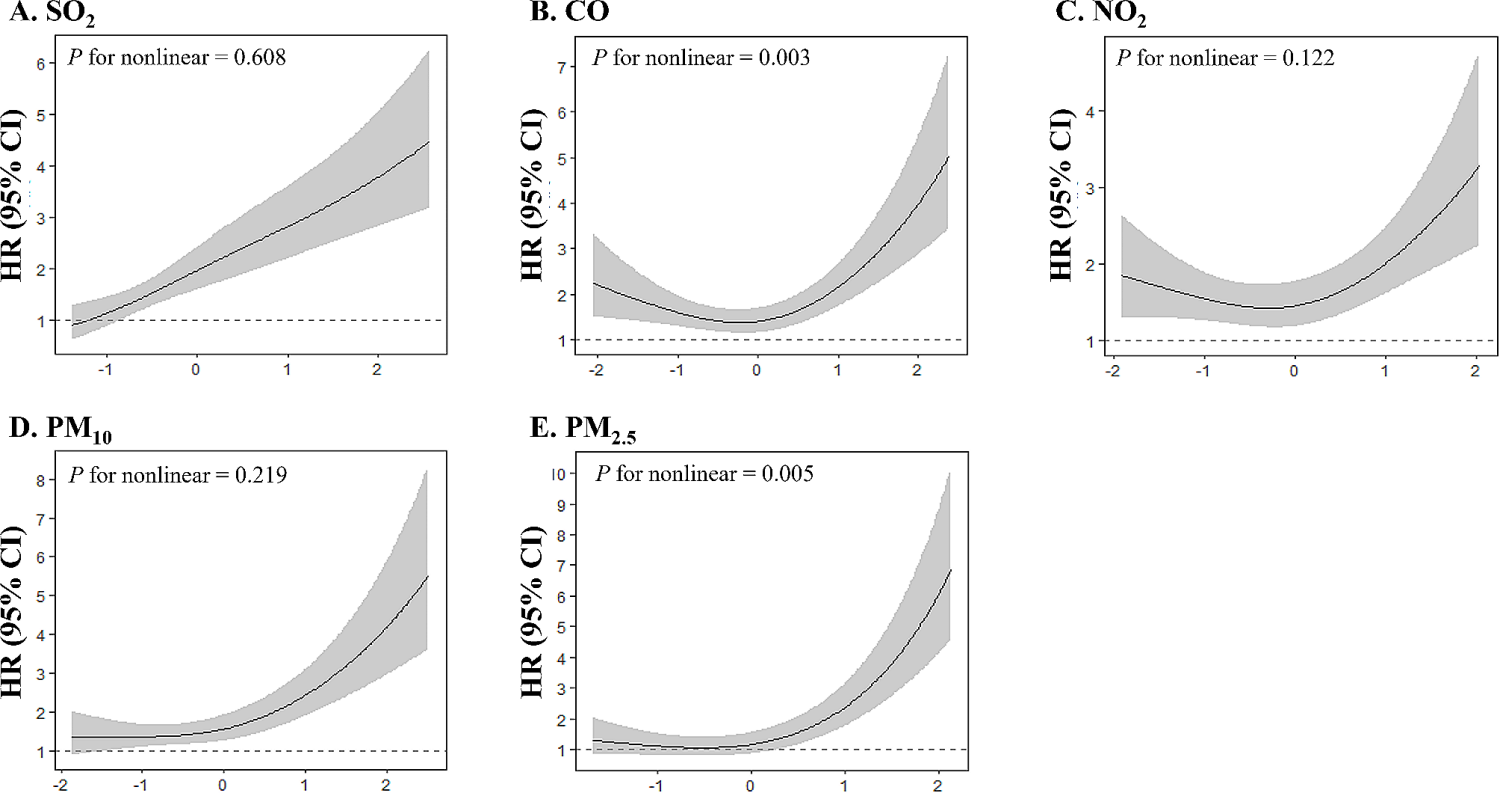
Restricted cubic spline regression analysis for malignant mesothelioma-related mortality risk with pollutant exposure levels. Hazard ratio (HR, solid lines) and 95% confidence interval (CI, gray area) for risk of malignant mesothelioma-related mortality along with the changes of standardized (A) SO_2_, (B) CO, (C) NO_2_, (D) PM_10_, and (E) PM_2.5_ exposure levels from the restricted cubic splines regression model. Models were adjusted for age at diagnosis, sex, smoking status, cancer cell type, type of treatment, asbestos exposure modalities, and month of diagnosis

**Fig. 2:**
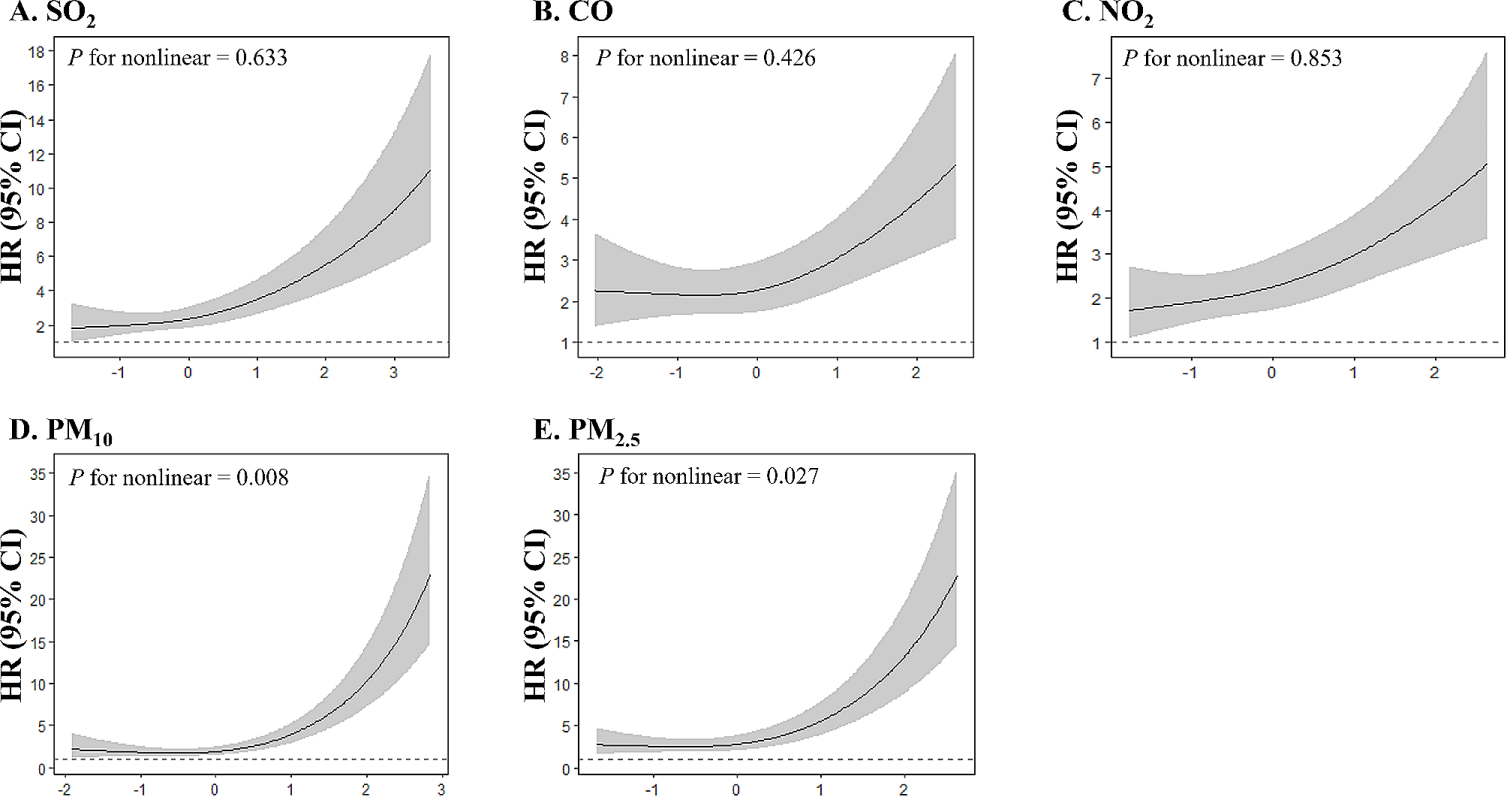
Restricted cubic spline regression analysis for the mortality risk of asbestos-related lung cancer with pollutant exposure levels. Hazard ratio (HR, solid lines) and 95% confidence interval (CI, gray area) for risk of asbestos-related lung cancer mortality along with the changes of standardized (A) SO_2_, (B) CO, (C) NO_2_, (D) PM_10_, and (E) PM_2.5_ exposure levels from the restricted cubic splines regression model. Models were adjusted for age at diagnosis, sex, smoking status, cancer cell type, type of treatment, asbestos exposure modalities, and month of diagnosis

Figures 1 and 2 show the restricted cubic spline analysis findings of HR variation with air pollutant exposure. We observed nonlinear associations between exposure and mortality for certain air pollutants. For malignant mesothelioma, the relationship between HR and both CO and PM_2.5_ exposures was nonlinear, whereas for lung cancer, the relationships of HR with PM_10_ and PM_2.5_ exposures were nonlinear. Steeper slopes were noted at above-average levels of CO, NO_2_, PM_10_, and PM_2.5_ exposures.

Figures S3 and S4 present the results of the two-pollutant model analysis of the association between air pollutant exposure and mortality. For malignant mesothelioma, the relationship between exposure to each air pollutant and the HR remained significantly positive, with the exception of CO exposure adjusted for PM_2.5_; NO_2_ exposure adjusted for SO_2_, CO, PM_10_, or PM_2.5_; and PM_2.5_ exposure adjusted for PM_10_ (Figure S3). Similarly, for lung cancer, the association between exposure to each air pollutant and the HR remained significantly positive, with the exception of CO exposure adjusted for PM_10_ or PM_2.5_ and NO_2_ exposure adjusted for PM_10_ or PM_2.5_ (Figure S4).

The results of the multi-pollutant model analysis using quantile g-computation are presented in Table 4. For malignant mesothelioma, a quartile increase in the mixture of SO_2_, CO, NO_2_, PM_10_, and PM_2.5_ was significantly associated with a 1.99-fold increase in the mortality risk (95% CI: 1.62, 2.44). The effect of the pollutant mixtures was primarily driven by SO_2_, followed by PM_10_ and PM_2.5_. For lung cancer, a quartile increase in the pollutant mixture was significantly associated with a 1.87-fold increase in the mortality risk (95% CI: 1.53, 2.30). The effect was primarily driven by PM_10_, followed by PM_2.5_ and SO_2_.

**Table 4:** Adjusted^a^ hazard ratios (HRs) and 95% confidence intervals (CIs) for cancer mortality associated with air pollution in multi-pollutant models from 1000 bootstrap replicates

Air pollutants	Beta	Effect of mixtures ln HR (95% CI)	HR (95% CI)
Malignant mesothelioma			
SO_2_	0.308	0.687 (0.484, 0.890)	1.99 (1.62, 2.44)
CO	0.041		
NO_2_	−0.219		
PM_10_	0.304		
PM_2.5_	0.253		
Lung cancer			
SO_2_	0.168	0.628 (0.423, 0.833)	1.87 (1.53, 2.30)
CO	0.053		
NO_2_	0.042		
PM_10_	0.192		
PM_2.5_	0.173		

^a^Models were adjusted for age at diagnosis, sex, smoking status, cancer cell type, type of treatment, asbestos exposure modalities, and month of diagnosis.

## Discussion

This study investigated the impact of air pollution on the survival of patients with malignant mesothelioma and asbestos-related lung cancer. The survival rates for both cancer types decreased with increasing exposure to all air pollutants, and the estimated HRs rose significantly with a 1-SD increase in each pollutant. Furthermore, a quartile increase in the mixture of pollutants was associated with an increased mortality risk from malignant mesothelioma and lung cancer.

Although the use of asbestos was banned in South Korea in 2009, it remains one of the countries where asbestos-related diseases continue to emerge due to the long latency period of asbestos. According to a report from the Environmental Health Center for Asbestos, the number of patients with asbestos-related cancer was 137 in 2011 (100 with malignant mesothelioma and 37 with lung cancer), but this number increased to 642 in 2022 (94 with malignant mesothelioma and 548 with lung cancer). Some studies, based on records of asbestos usage in South Korea, have estimated the mortality rate due to asbestos-related diseases [24–26], predicting a peak in the 2020s when considering a latency period of 33 years [24]. However, the duration of emerging cases may extend further given the possibility of a longer latency period for certain diseases [27], signifying an increased number of patients requiring health management at the national level. While numerous studies have been conducted in South Korea on the epidemiological characteristics of patients and the association with exposure sources [28–32], strategies for improving the conditions of asbestos victims remain unclear. Our findings suggest that reducing air pollution could ameliorate the prognosis of patients with malignant mesothelioma and lung cancer, potentially reducing the mortality risk, thereby improving the condition of asbestos victims.

Despite the known health effects of air pollution, few studies have investigated its influence on survival time after cancer diagnosis. For malignant mesothelioma, excluding studies on treatments, most prior studies have focused on examining the impact of demographic characteristics [21, 33–36], asbestos exposure patterns [21, 34], and personal habits such as smoking on survival rates [33, 37]. However, three studies have investigated the contribution of air pollution to survival after lung cancer diagnosis. Between 1992 and 2008, Xu et al. [38] examined patients with respiratory cancers from Honolulu and Los Angeles and found that the HRs for cancer-specific mortality were 1.43 (95% CI: 1.39, 1.46), 1.49 (95% CI: 1.45, 1.53), and 1.06 (95% CI: 1.04, 1.07) for a 10-µg/m^3^ increase in PM_10_, 5-µg/m^3^ increase in PM_2.5_, and 10-ppb increase in O_3_, respectively. Eckel et al. [39] and McKeon et al. [40] reported that increased average annual exposures to NO_2_, O_3_, PM_10_, and PM_2.5_ were associated with a heightened mortality risk in patients with lung cancer. Our study was distinct in that it additionally considered asbestos exposure; nevertheless, our findings were consistent with those of previous studies.

The pathophysiological mechanisms underlying the relationship between air pollution and the survival of individuals with malignant mesothelioma and lung cancer are uncertain; however, several plausible explanations exist for the abovementioned pathophysiological mechanism. First, air pollution exposure is suggested to induce oxidative stress, leading to DNA damage. SO_2_ generates reactive oxygen species (ROS), inducing oxidative stress in various organs [41]. Nitrogen pollutants potentiate the effect of oxidative stress on the progression and development of various cancers [42]. Additionally, PM exposure generates ROS, promoting oxidative stress [18]. Oxidative stress in cancer cells significantly affects survival time by promoting cell growth, genetic instability, and mutations [43]. Second, inflammation resulting from air pollution exposure is another potential pathophysiological mechanism. Zhang et al. [44], via a cohort panel study, observed increased plasma levels of IL-6, a proinflammatory cytokine, in elderly residents of the Los Angeles metropolitan area exposed to nitrogen oxides (NOx) and PM. Inflammation primarily induces DNA damage through the effect of ROS and reactive nitrogen species, and unrepairable damage may facilitate cancer development [45]. Therefore, air pollution-induced inflammation may accelerate cancer-related mortality. Lastly, air pollution exposure is associated with the activation of nuclear factor-erythroid 2-related factor 2 (NRF2). The NRF2 pathway is a transcription pathway that regulates the expression of several key genes involved in antioxidant enzymes and redox homeostasis, playing a vital role in modulating the intracellular redox environment. An excessive activation of the NRF2 pathway can promote tumor formation and chemotherapeutic drug resistance [46–48]. Exposure to air pollutants, including NOx and carbon monoxide, has been reported to activate the NRF2 pathway [11], and PM_2.5_ has been demonstrated to increase NRF2 expression levels in mice with chronic obstructive pulmonary disease [49].

The dose-response relationship observed in our study between air pollution exposure and mortality closely resembles that of a J-shaped curve. Such a pattern has been commonly observed in previous studies investigating the health effects of air pollution. Studies examining the effects of air pollution on the survival of patients with lung cancer in California and Pennsylvania have shown associations of NO_2_, PM_10_, and PM_2.5_ exposures with mortality in a J- or U-shaped manner [39, 40]. In a study using a national Medicare cohort from 2000 to 2016, the dose-response relationship between chronic NO_2_ exposure and the relative risk of mortality was close to that of a J-shaped curve [50]. Similar findings have been confirmed in animal experiments, where dose-response relationships in both experiments observing lung tumors in rats exposed to various types of PM and lung inflammation in mice exposed to ultra fine carbon particles exhibited J-shaped patterns, and the authors interpreted the results as indicative of a threshold level of exposure [51, 52]. Cox Jr [53] explained these dose-response relationships with the hypothesis of hormesis and argued that the relationship between PM exposure and mortality risk may not be linear. If air pollution exposure indeed exhibits a J-shaped dose-response relationship as observed in the abovementioned studies, it may be possible to improve the survival time of patients with asbestos-related cancer by maintaining their exposure to air pollution below a certain level. Therefore, the dose-response relationship between air pollution exposure and mortality in patients with cancer warrants further investigation.

Using quantile-based g-computation, we identified statistically significant adverse effects of air pollutant mixtures on both all-cause and cancer-specific mortalities. Considering that the single effects of all air pollutants were most prominently observed in the two-pollutant model, no positive interaction effect was found among SO_2_, CO, NO_2_, PM_10_, and PM_2.5_ exposures in this study. Li et al. [54] utilized Bayesian kernel machine regression to observe adverse effects of air pollutant mixtures (PM_2.5_, O_3_, and NO_2_) on overall mortality in the United States Medicare population; however, they did not find interaction effects among the substances, which was consistent with our findings. Meanwhile, the mixture exposure effects observed in our study were primarily driven by SO_2_, PM_10_, and PM_2.5_, and the effect of NO_2_ exposure became non-significant after adjusting for PM exposure. Huang et al. [55] reported that PM_2.5_ exposure yielded the largest contribution to increased mortality risk, followed by SO_2_ and PM_10_ exposures, and Ji et al. [56] found that the HR for NO_2_-related mortality in a Chinese elderly population became negligible after adjusting for PM_2.5_ exposure; these findings were consistent with our study findings. Based on this cumulative evidence, PM and SO_2_ exposure levels should be reduced to improve patient survival, rather than worrying about synergy among air pollutants. However, there is limited evidence regarding the relative contributions of air pollution mixtures to mortality [57, 58], necessitating further research in this area.

Our study has several limitations. First, the nationwide atmospheric monitoring data used in this study may not precisely reflect the actual air pollution exposure levels for patients. Patients with cancer are likely to spend more time indoors compared with individuals without cancer. In addition, misclassification of air pollutant exposures could occur if patients sought medical services in different locations for better healthcare. Second, our study did not consider the educational and income levels of patients. Although the initial questionnaire included these parameters, they were later excluded from subsequent surveys because most respondents refused to provide information about their educational and income levels. Some prior large-sample studies evaluating the impact of air pollution on mortality in patients with lung cancer have attempted to address these issues by quantifying the socioeconomic status of regions as covariates [39, 40]. In this study, we presented supplementary data that included analysis adjusted for quartiles of average educational and income levels by administrative regions (Table S2). However, no significant changes were observed in the existing results. Additionally, classifying study participants by region would result in a considerable small sample size, making it difficult to adequately represent each region by study participants. Future studies should obtain additional data to properly account for the socioeconomic level of surveyed participants.

## Conclusions

This study found evidence that exposure to air pollution adversely affects the survival time of patients with malignant mesothelioma and asbestos-related lung cancer. There were no observed interaction effects among air pollutants, and the effects of the pollutant mixture were primarily driven by SO_2_, PM_10_, and PM_2.5_. Despite the low survival rates among patients with asbestos-related cancer, approaches controlling for environmental factors have been scarcely considered. Therefore, the findings of this study could have important public health implications for patient survival.

### Electronic supplementary material

Below is the link to the electronic supplementary material.

Supplementary Material 1

## Data Availability

The data that support the findings of this study are available from the Ministry of Environment but restrictions apply to the availability of these data, which were used under license for the current study, and so are not publicly available. Data are however available from the authors upon reasonable request and with permission of the Ministry of Environment.
