# Supplementary material for: Air pollution and survival in patients with malignant mesothelioma and asbestos-related lung cancer: a follow-up study of 1591 patients in South Korea
Source: Environ Health. 2024 Jun 10;23:56. doi: 10.1186/s12940-024-01094-y (PMC11163745; doi:10.1186/s12940-024-01094-y)
Supplement: Supplementary file 1 — Supplementary Material 1 [file 12940_2024_1094_MOESM1_ESM.docx]

**Supplementary materials**

**Air pollution and Survival in Patients with Malignant Mesothelioma and Asbestos-related Lung Cancer: A Follow-up Study of 1591 Patients in South Korea**

Da-An Huh^a,*^, Yun-Hee Choi^b^, Lita Kim^c,d^, Kangyeon Park^c,d^, Jiyoun Lee^e^, Se Hyun Hwang^e^, Kyong Whan Moon^d,e^, Min-Sung Kang^f,*^, Yong-Jin Lee^f,g^

^a^Institute of Health Sciences, Korea University, Anam-ro 145, Seongbuk-gu, Seoul 02841, South Korea

^b^Department of Ophthalmology, Korea University College of Medicine, Anam-ro 145, Seongbuk-gu, Seoul 02841, South Korea

^c^Department of Health and Safety Convergence Science, Korea University, Anam-ro 145, Seongbuk-gu, Seoul 02841, South Korea

^d^BK21 FOUR R & E Center for Learning Health System, Korea University, Anam-ro 145, Seongbuk-gu, Seoul 02841, South Korea

^e^School of Health and Environmental Science, Korea University, Anam-ro 145, Seongbuk-gu, Seoul 02841, South Korea

^f^Asbestos Environmental Health Center, Soonchunhyang University Cheonan Hospital, 31 Suncheonhyang 6-gil, Dongnam-gu, Cheonan-si 31151, South Korea

^g^Department of Occupational & Environmental Medicine, Soonchunhyang University, 31 Suncheonhyang 6-gil, Dongnam-gu, Cheonan-si 31151, South Korea

Table S1. Median survival time and survival rate by changes in covariate categories.

| Categorized air pollution exposure | Malignant mesothelioma (n=593) | | | | Lung cancer (n=998) | | | |
| --- | --- | --- | --- | --- | --- | --- | --- | --- |
|  | Median  survival (years) | Survival rate (%) | | | Median  survival (years) | Survival rate (%) | | |
|  |  | 1-year | 3-year | 5-year |  | 1-year | 3-year | 5-year |
| Age at diagnosis |  |  |  |  |  |  |  |  |
| ≤50 | 2.42 | 82.7 | 53.1 | 50.6 | 3.33 | 98.2 | 94.6 | 87.5 |
| 50–60 | 2.17 | 77.7 | 48.2 | 38.1 | 3.17 | 96.7 | 86.8 | 83.5 |
| 60–70 | 1.33 | 69.9 | 38.8 | 28.6 | 2.67 | 93.2 | 79.3 | 73.2 |
| >70 | 1.08 | 58.8 | 31.6 | 22.0 | 1.83 | 80.9 | 60.5 | 52.6 |
| Sex |  |  |  |  |  |  |  |  |
| Male | 1.33 | 65.9 | 33.2 | 25.8 | 2.50 | 87.3 | 69.5 | 63.0 |
| Female | 1.79 | 76.7 | 52.6 | 41.4 | 2.75 | 96.2 | 88.0 | 82.8 |
| Year of diagnosis, % |  |  |  |  |  |  |  |  |
| 2009–2012 | 1.92 | 70.0 | 28.6 | 20.7 | 7.58 | 98.2 | 94.6 | 87.5 |
| 2013–2015 | 1.67 | 64.4 | 37.8 | 24.4 | 7.00 | 96.7 | 86.8 | 83.5 |
| 2016–2018 | 2.13 | 71.7 | 42.0 | 27.5 | 4.42 | 93.2 | 79.3 | 73.2 |
| 2019–2022 | 1.08 | 73.3 | 51.7 | 49.4 | 1.75 | 80.9 | 60.5 | 52.6 |
| Smoking status, % |  |  |  |  |  |  |  |  |
| Never smoked | 1.75 | 73.7 | 48.5 | 37.5 | 3.21 | 94.3 | 83.0 | 76.9 |
| Past smoker | 1.33 | 66.7 | 32.9 | 25.5 | 2.33 | 88.0 | 69.5 | 63.3 |
| Current smoker | 2.00 | 57.1 | 28.6 | 28.6 | 1.63 | 80.0 | 70.0 | 70.0 |
| Unknown | 1.29 | 68.4 | 36.8 | 31.6 | 1.92 | 85.9 | 81.3 | 76.6 |
| Asbestos exposure  modalities, % |  |  |  |  |  |  |  |  |
| Environmental | 1.75 | 74.7 | 47.7 | 39.5 | 2.58 | 91.7 | 79.4 | 73.0 |
| Occupational | 1.50 | 65.9 | 33.6 | 22.9 | 2.25 | 89.3 | 73.3 | 68.7 |
| Co-exposure | 1.08 | 64.0 | 33.3 | 26.7 | 2.83 | 89.9 | 74.1 | 68.0 |
| Surgery, % |  |  |  |  |  |  |  |  |
| No | 1.33 | 66.8 | 35.3 | 26.8 | 2.25 | 83.4 | 60.3 | 52.4 |
| Yes | 2.25 | 78.4 | 54.4 | 44.4 | 3.33 | 98.3 | 93.5 | 89.6 |
| Radiation, % |  |  |  |  |  |  |  |  |
| No | 1.58 | 70.0 | 40.5 | 31.6 | 2.58 | 90.9 | 78.3 | 72.6 |
| Yes | 2.83 | 80.0 | 60.0 | 50.0 | 2.33 | 88.3 | 63.5 | 55.5 |
| Chemotherapy, % |  |  |  |  |  |  |  |  |
| No | 1.50 | 70.6 | 45.4 | 35.5 | 2.67 | 90.9 | 81.0 | 75.8 |
| Yes | 1.58 | 69.8 | 36.7 | 28.6 | 2.58 | 90.0 | 69.2 | 61.9 |
| Cancer cell type  (malignant mesothelioma) |  |  |  |  |  |  |  |  |
| Epithelioid | 2.00 | 76.0 | 48.7 | 37.3 | - | - | - | - |
| Sarcomatoid | 0.75 | 46.4 | 18.8 | 17.4 | - | - | - | - |
| Biphasic | 1.17 | 65.0 | 26.2 | 21.4 | - | - | - | - |
| Cancer cell type  (lung cancer) |  |  |  |  |  |  |  |  |
| Adenocarcinoma | - | - | - | - | 2.83 | 95.2 | 84.1 | 78.5 |
| Squamous cell | - | - | - | - | 2.33 | 87.7 | 70.8 | 64.8 |
| Small cell | - | - | - | - | 1.42 | 78.9 | 54.9 | 49.3 |
| Large cell | - | - | - | - | 3.50 | 77.8 | 66.7 | 55.6 |
| Other | - | - | - | - | 2.42 | 76.7 | 54.7 | 45.3 |

Table S2. Adjusted^a^ hazard ratios (HRs) and 95% confidence intervals (CIs) for all-cause and cancer-specific mortalities associated with a 1-standard deviation (SD) increase in air pollutant exposure^b^, further adjusted for surrogate indicators of educational and income levels

| Air pollutant | Malignant mesothelioma (n=593) | | Lung cancer (n=998) | |
| --- | --- | --- | --- | --- |
|  | All-cause mortality HR (95% CI) | Cancer-specific mortality HR (95% CI) | All-cause mortality HR (95% CI) | Cancer-specific mortality HR (95% CI) |
| SO_2_ | 1.43 (1.29, 1.58) | 1.44 (1.30, 1.59) | 1.40 (1.24, 1.56) | 1.42 (1.27, 1.61) |
| CO | 1.27 (1.12, 1.44) | 1.25 (1.11, 1.43) | 1.20 (1.08, 1.35) | 1.22 (1.10, 1.37) |
| NO_2_ | 1.13 (1.01, 1.28) | 1.10 (0.99, 1.25) | 1.27 (1.14, 1.42) | 1.28 (1.16, 1.43) |
| PM_10_ | 1.51 (1.31, 1.73) | 1.45 (1.26, 1.66) | 2.01 (1.75, 2.31) | 2.05 (1.77, 2.36) |
| PM_2.5_ | 1.70 (1.43, 2.00) | 1.64 (1.39, 1.95) | 1.90 (1.65, 2.19) | 1.93 (1.68, 2.22) |

^a^Models were adjusted for age at diagnosis, sex, smoking status, cancer cell type, type of treatment, asbestos exposure modalities, month of diagnosis, and educational and income levels by administrative regions. For the education level variable, we used the proportion of residents with at least a high school diploma, as provided by the Korean Statistical Information Service (KOSIS), and applied quartiles based on the average values for each administrative region. For the income variable, we used the average income values for each administrative region provided by the Korea Culture Information Service Agency (KCISA), and also applied quartiles based on these average values.

^b^SD values: For malignant mesothelioma, 1.51 ppb (SO_2_), 102.20 ppb (CO), 7.33 ppb (NO_2_), 9.24 µg/m^3^ (PM_10_), and 4.76 µg/m^3^ (PM_2.5_). For lung cancer, 1.01 ppb (SO_2_), 79.51 ppb (CO), 5.86 ppb (NO_2_), 7.43 µg/m^3^ (PM_10_), and 4.38 µg/m^3^ (PM_2.5_)


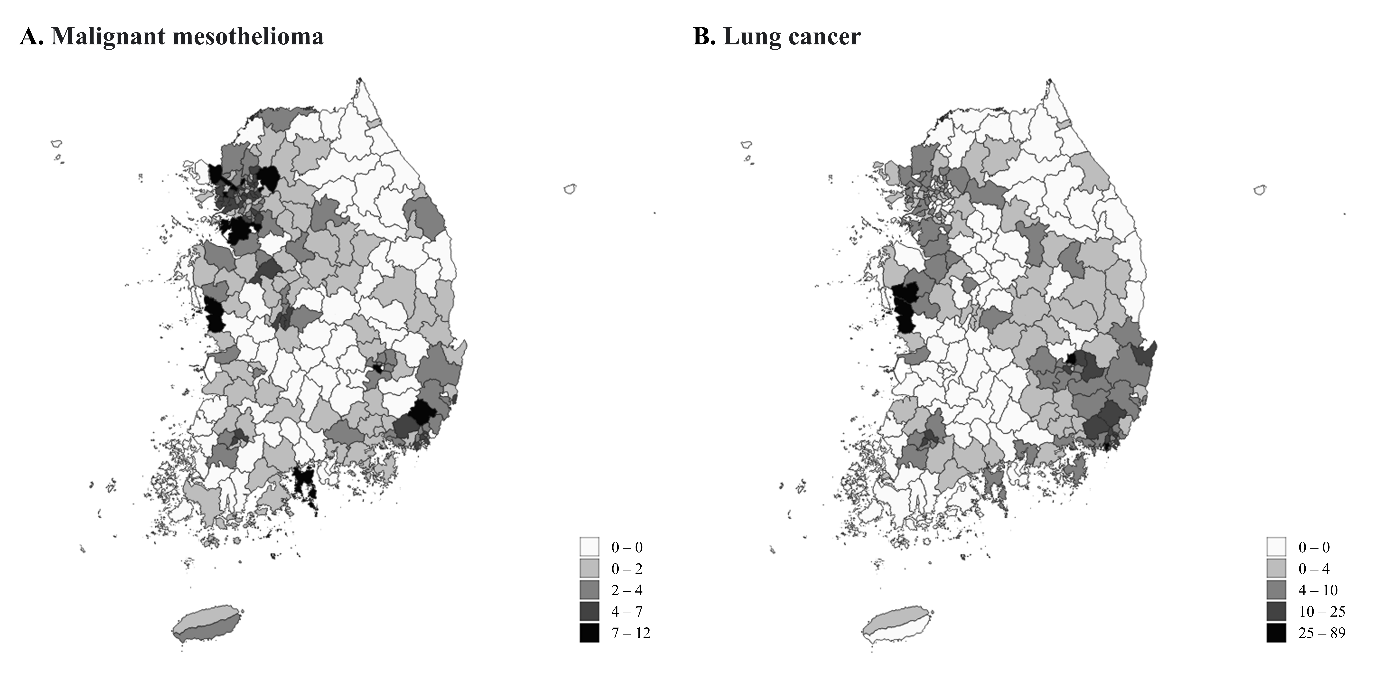


Figure S1. Geometric distribution of patients with malignant mesothelioma and asbestos-related lung cancer by administrative region.


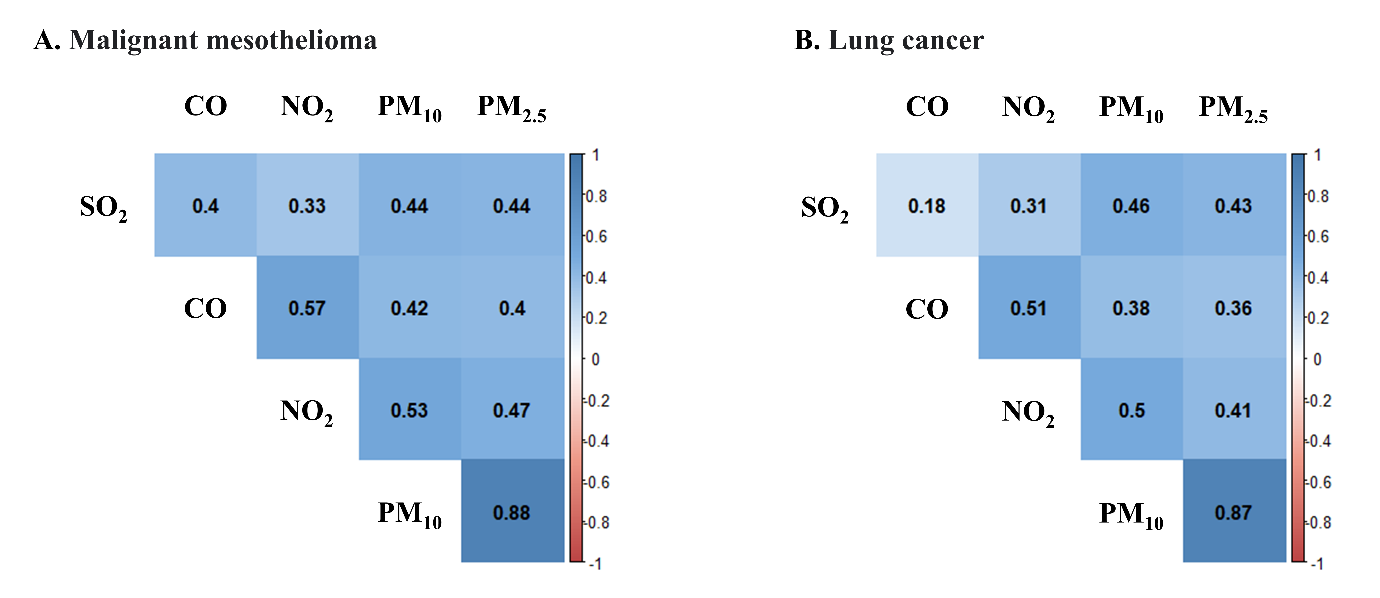


Figure S2. Pearson correlations among air pollutants.


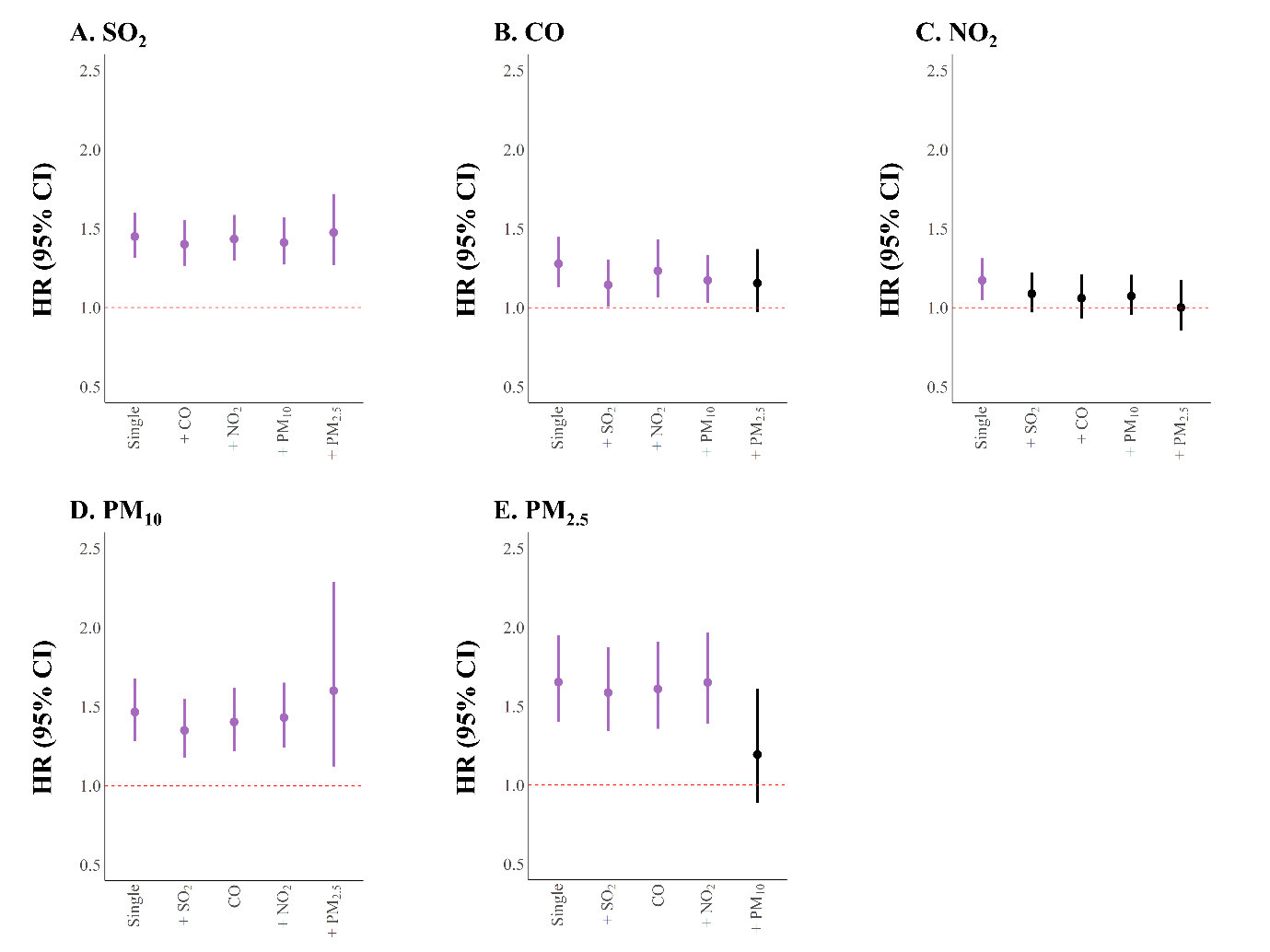


Figure S3. Associations of a 1 standard deviation (SD) increase in (A) SO_2_, (B) CO, (C) NO_2_, (D) PM_10_, and (E) PM_2.5_ exposure with death of malignant mesothelioma in two-pollutant models. Models were adjusted for age at diagnosis, sex, smoking status, cancer cell type, type of treatment, asbestos exposure modalities, and month of diagnosis. Associations with statistical significance were shown in purple color.


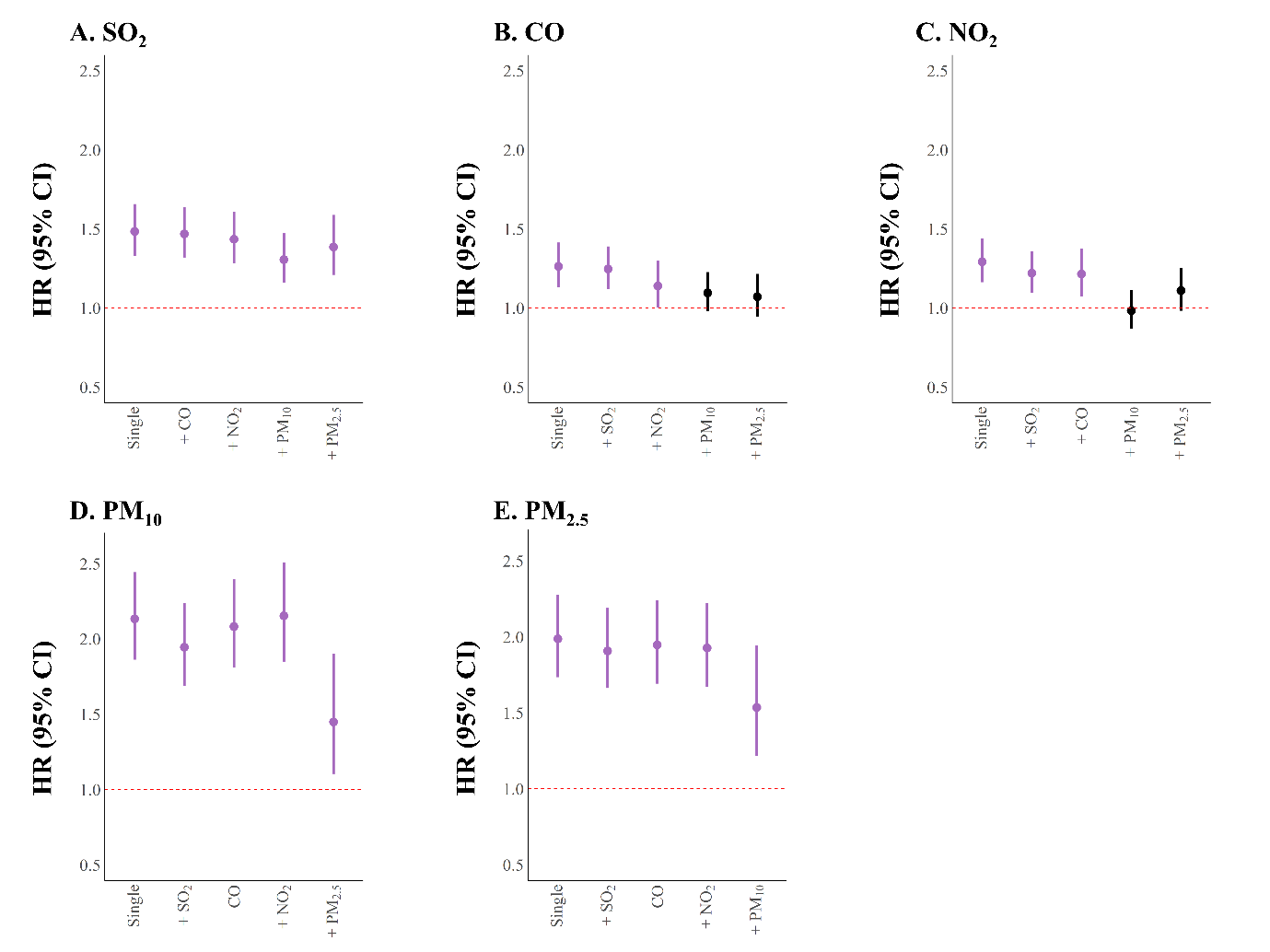


Figure S4. Associations of a 1 standard deviation (SD) increase in (A) SO_2_, (B) CO, (C) NO_2_, (D) PM_10_, and (E) PM_2.5_ exposure with death of asbestos-related lung cancer in two-pollutant models. Models were adjusted for age at diagnosis, sex, smoking status, cancer cell type, type of treatment, asbestos exposure modalities, and month of diagnosis. Associations with statistical significance were shown in purple color.
